# Solitary Bone Plasmacytoma Progressing into Retroperitoneal Plasma Cell Myeloma with No Related End Organ or Tissue Impairment: A Case Report and Review of the Literature

**DOI:** 10.4274/Tjh.2012.0167

**Published:** 2014-09-05

**Authors:** Gargi Tikku, Monica Jain, Asit Mridha, Rajesh Grover

**Affiliations:** 1 Delhi State Cancer Institute, Department of Oncopathology, Delhi, India; 2 Delhi State Cancer Institute, Department of Radiotherapy, Delhi, India

**Keywords:** Solitary bone plasmacytoma, Retroperitoneal plasma cell myeloma, Related end organ or tissue impairment, Bone marrow plasmacytosis

## Abstract

Solitary bone plasmacytomas and plasma cell myeloma are clonal proliferations of plasma cells. Many patients with solitary bone plasmacytomas develop plasma cell myeloma on follow-up. We present a case of a 70-year-old man who presented with fracture and a lytic lesion in the subtrochanteric region of the left femur and was assigned a diagnosis of solitary bone plasmacytoma. He received local curative radiotherapy. However, 4 months later his serum M protein and β2-microglobulin levels increased to 2.31 g/dL and 5.965 mg/L, respectively. He complained of abdominal fullness and constipation. Ultrasound and non-contrast CT imaging revealed multiple retroperitoneal masses. Colonoscopic examination was normal. Biopsy of the a retroperitoneal mass confirmed it to be a plasmacytoma. Repeat hemogram, blood urea, serum creatinine, skeletal survey, and bone marrow examination revealed no abnormalities. This is an unusual presentation of plasma cell myeloma, which manifested as multiple huge extramedullary retroperitoneal masses and arose from a solitary bone plasmacytoma, without related end organ or tissue impairment and bone marrow plasmacytosis. The patient succumbed to his disease 8 months after the appearance of the retroperitoneal masses. This case highlights the importance of close monitoring of patients diagnosed with solitary bone plasmacytoma with increased serum M protein and serum β2-microglobulin levels, so that early therapy can be instituted to prevent conversion to plasma cell myeloma.

## OZET

Soliter kemik plazmasitomları (SKP) ve plazma hücreli miyelom (PHM) plazma hücrelerinin klonal proliferatif hastalıklarıdır. SKP’si olan hastaların çoğu izlemde PHM geliştirir. Burada sol femurda subtrokanterik bölgede kırık ve litik lezyonla başvuran ve SKP tanısı alan 70 yaşındaki erkek hasta sunulmuştur. Hastaya lokal küratif radyoterapi uygulanmıştır. Ancak 4 ay sonrasında hastanın serum M proteini ve β2-mikroglobulin düzeyleri sırasıyla 2,31 g/dL ve 5,965 mg/L düzeylerine çıkmıştır. Hastanın eşlik eden karında şişkinlik ve kabızlık yakınmaları bulunmaktadır. Kolonoskopik incelemesi normal olan hastanın retroperitoneal kitlesinden alınan biyopsi sonucunda plazmasitoma tanısı doğrulanmıştır. Tekrarlanan tam kan sayımı, kan üre, serum kreatinin, kemik taraması ve kemik iliği incelemelerinde anormallik bulunmamıştır. Çok sayıda ekstramedüller retroperitoneal kitlelerle bulgu vermesi, SKP gelişmesi, organ ya da doku hasarlanmasına neden olmaması ve kemik iliği tutulumunun bulunmaması gibi nedenlerle özellikli bir PHM olgusu sunulmuştur. Hasta retroperitoneal kitleler belirdikten 8 ay sonra kaybedilmiştir. Olgumuz, SKP tanısı alan ve yüksek serum M proteini ve β2-mikroglobulin düzeylerine sahip hastaların yakın izlenmesi gerektiğine ve PHM dönüşümü önlemek amacıyla erken tedavi başlanmasının uygun olabileceğine işaret etmektedir.

## INTRODUCTION

Solitary bone plasmacytomas (SBPs) and plasma cell myeloma (PCM) are clonal proliferations of plasma cells. The majority of patients with apparent SBP go on to develop PCM and approximately 5% of all patients with PCM have an initial diagnosis of solitary plasmacytoma [1]. Here we present a case of SBP progressing to PCM with unusual features.

## CASE PRESENTATION

**Figure 1:**
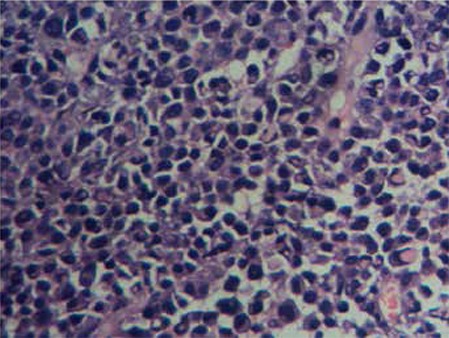
H&E section showing retroperitoneal plasmacytoma (40x).

A 70-year-old male came to the hospital with fracture of the left femur. X-ray of the left femur showed a lytic lesion in the subtrochanteric region. Laboratory investigations revealed hemoglobin level of 10.2 g/dL, white blood cell count of 8200/µL, blood urea of 21 mg/dL, creatinine of 1.1 mg/dL, serum Ca2+ of 9.1 mg/dL, total protein of 7.8 g/dL, and albumin of 3.01 g/dL (A:G=0.63). Open reduction and internal fixation were done using a dynamic hip fixator, but there was no healing of the fracture even after 5 months. A biopsy from the fracture site showed sheets of CD138-positive plasma cells with kappa light chain restriction. Bone marrow was normocellular with normal hemopoietic cells and plasma cells constituted 2% of the nucleated differential count. Serum β2-microglobulin was 6.3 ng/L and M spike was 0.74 g/dL. Immunofixation electrophoresis revealed the M spike in the β-globulin region to be of IgA kappa. Bence Jones proteins were absent in the urine. No other lytic or soft tissue lesions were detected in a whole-body MRI scan. Hence, a diagnosis of SBP was made. The patient received radiotherapy (26 cycles) to the left femur over a period of 1 month with a total dose of 5000 cGy. Four months after completion of radiotherapy, a repeat serum protein electrophoresis showed an M spike measuring 2.31 g/dL in the β-globulin region, β2-microglobulin of 5.965 mg/L, total protein of 8.6 g/dL, albumin of 3.2 g/dL, and an A:G ratio of 0.59. The patient complained of diffuse abdominal pain, swelling, and constipation. Abdominal ultrasound revealed a heterogeneous, hypervascular mass of 9.4x6.2 cm in the left suprarenal area, displacing the pancreas anteriorly, and large peripancreatic lymph nodes. Non-contrast CT imaging showed a mass (6.6x4.5 cm) near the pylorus with multiple peripancreatic lymph nodes and a left suprarenal retroperitoneal mass. Colonoscopic examination was normal. Tru-Cut biopsy from the retroperitoneal mass revealed extramedullary plasmacytoma (Figure 1). Blood analysis showed a hemoglobin level of 10.8 g/dL, serum calcium of 8.1 mg/dL, and creatinine of 1.09 mg/dL. Urine analysis did not reveal any Bence Jones proteins. Bone marrow aspirate showed 2% plasma cells. Bone marrow biopsy showed no increase or clusters of plasma cells. A whole-body PET-CT scan showed multiple metabolically active mass lesions in the retroperitoneum and peripancreatic lymph nodes. The uptake in the lungs, liver, spleen, remainder of the lymph node group regions, and skeletal system was within normal limits. A diagnosis of PCM was made. The patient was treated with chemotherapy with subcutaneous injection of bortezomib and dexamethasone. There was a decrease in the size of the mass after chemotherapy, but the patient’s condition deteriorated and he died 2 months after initiation of chemotherapy.

## DISCUSSION AND REVIEW OF THE LITERATURE

Solitary plasmacytoma, a localized collection of monoclonal plasma cells without evidence of a systemic plasma cell proliferative disorder (e.g., PCM), constitutes less than 3%-5% of all plasma cell neoplasms. There are 2 types of plasmacytomas: 1) solitary plasmacytoma of the bone and 2) extraosseous (extramedullary) plasmacytoma [2,3]. SBP is more common in men (65%), the median age at diagnosis being 55 years. The most commonly affected bones in order of frequency are the vertebrae, ribs, skull, pelvis, femur, clavicle, and scapula [4]. The thoracic vertebrae are commonly involved and long bone involvement below the elbow or knee is rare [3,5].

Recommended diagnostic criteria for SBP [4] include: 1) a single area of bone destruction due to clonal plasma cells; 2) normal marrow without clonal disease; 3) normal results upon skeletal survey and MRI imaging of the spine, pelvis, proximal femora, and humeri; 4) no anemia, hypercalcemia, or renal impairment attributable to myeloma; and 5) absent or low serum or urinary level of monoclonal protein and preserved levels of uninvolved immunoglobulins.

Our case fulfilled all of the recommended diagnostic criteria for SBP. Biopsy from the left femur lesion demonstrated monoclonal CD138-positive plasma cells with kappa light chain restriction without related end organ or tissue impairment (RETI) or bone marrow plasmacytosis. Serum protein electrophoresis showed an M spike of 0.74 g/dL in the β2 region and serum immunofixation electrophoresis confirmed the M spike to be of IgA kappa. M protein in the serum or urine has been noted in 24%-72% of SBP patients in various series [4,6,7] with normal uninvolved immunoglobulin levels. In our case, initial plasma proteins were normal and Bence Jones protein was absent.

The preferred treatment for SBP is curative radiotherapy (>4000 cGy), which can give long-term disease-free survival in approximately 30% of cases [8]. Our patient also received radiotherapy to the left femur with curative intent, but failed to respond. Repeat serum protein evaluation revealed a rising M spike in the β2 region (2.31 g/dL), which indicated disease progression. The patient had abdominal fullness, constipation, and multiple retroperitoneal masses. There was reversal of the A:G ratio with increasing globulin levels. Urinary Bence Jones protein remained absent. Histopathological examination of the retroperitoneal masses confirmed extramedullary plasmacytoma. The serum β2-microglobulin level also increased from 6.3 ng/L to 5.965 mg/L, denoting Stage III PCM according to the current International Staging System [9].

SBPs are known to progress to PCM in a majority of patients [4,8,10]. Bataille et al. [11], in their study of 114 cases of solitary myeloma, revealed that 85% of the patients experienced disease progression after 10 years of diagnosis. Among the remaining 15% of patients without progression at 10 years, the monoclonal protein always disappeared following treatment with surgery and/or radiation therapy. Additionally, Wilder et al. [12] found that patients with complete disappearance of paraprotein by 1 year were relatively stable as compared to patients with persistence of paraprotein after 1 year, indicating the absence of occult disease outside the radiotherapy port. It has been observed that increasing levels of M protein or failure to completely remove the M protein from the serum and/or urine after effective local radiotherapy in SBP is associated with progression to multiple myeloma, as was seen in our case. Thus, serial quantification of M protein allows easy assessment of the course of disease and efficacy of treatment [13].

The serum β2-microglobulin level was significant; however, no renal impairment was present in this patient throughout the course of the disease. These markedly increased levels of β2-microglobulin reflected the appearance of huge retroperitoneal masses with increasing tumor burden and conversion to PCM.

Normal renal function may be related to the absence of secretion of Bence Jones proteins in the urine, which are known to cause renal tubular damage. Another cause for renal damage is hypercalcemia, which by inducing vasoconstriction followed by a decrease in glomerular filtration rate may also lead to renal damage; however, there was no hypercalcemia in our case as there were no multiple lytic bony lesions.

PCM is a bone marrow-based, multifocal plasma cell neoplasm associated with M protein in the serum and/or urine. In most cases there is generalized bone marrow involvement. The most common sites are in the bone marrow areas of the most active hematopoiesis. Extramedullary involvement is generally a manifestation of advanced disease [2]. In our case, there was no bone marrow plasmacytosis even when the patient developed Stage III PCM.

The unusual feature of disease progression in our case manifested as multiple huge extramedullary retroperitoneal masses, which have not been reported before in the literature. Only a few cases of retroperitoneal plasmacytomas have been reported to date [14,15,16,17,18,19,20,21]. All of these were primary retroperitoneal tumors (extramedullary plasmacytomas) with no bony lesions on skeletal survey. In all of these cases, bone marrow aspiration and biopsy were normal.

About 8% of patients with PCM are initially asymptomatic [22]. The majority have between 10% and 20% bone marrow plasma cells and the median level of serum M protein is nearly 30 g/L. Normal polyclonal immunoglobulins are reduced in >90% of patients and approximately 70% have monoclonal light chains in the urine [23]. The unique features in our case were the low serum M protein (i.e. below 30 g/L), the critical value of PCM, and the absence of RETI. 

With conversion to PCM, the patient received chemotherapy with bortezomib and dexamethasone, which led to a decrease in the size of the mass. However, the patient succumbed to his disease 2 months after initiation of chemotherapy and 11 months after the diagnosis of SBP. 

## CONCLUSIONS

1. Rising M protein in serum after local curative radiotherapy doses calls for a more radical treatment approach in the form of chemotherapy, even if the disease is localized to bone with no evidence of RETI.

2. Rising β2-microglobulin levels indicate disease progression even when there is no evidence of RETI.

3. Bone marrow plasmacytosis is not necessary for the diagnosis of PCM.

**Conflict of Interest Statement**

The authors of this paper have no conflicts of interest, including specific financial interests, relationships, and/ or affiliations relevant to the subject matter or materials included.
